# A New Distribution Family for Microarray Data †

**DOI:** 10.3390/microarrays6010005

**Published:** 2017-02-10

**Authors:** Diana Mabel Kelmansky, Lila Ricci

**Affiliations:** 1 Instituto de Cálculo, UBA-CONICET, Buenos Aires, Argentina; 2 Centro Marplatense de Investigaciones Matemáticas, UNMdP, Mar del Plata, Argentina; lricci@mdp.edu.ar

**Keywords:** data analysis, microarrays, *gpower*-normal, pseudo-dispersion models, truncated normal, combined maximum likelihood estimators

## Abstract

The traditional approach with microarray data has been to apply transformations that approximately normalize them, with the drawback of losing the original scale. The alternative standpoint taken here is to search for models that fit the data, characterized by the presence of negative values, preserving their scale; one advantage of this strategy is that it facilitates a direct interpretation of the results. A new family of distributions named *gpower*-normal indexed by p∈R is introduced and it is proven that these variables become normal or truncated normal when a suitable *gpower* transformation is applied. Expressions are given for moments and quantiles, in terms of the truncated normal density. This new family can be used to model asymmetric data that include non-positive values, as required for microarray analysis. Moreover, it has been proven that the *gpower*-normal family is a special case of pseudo-dispersion models, inheriting all the good properties of these models, such as asymptotic normality for small variances. A combined maximum likelihood method is proposed to estimate the model parameters, and it is applied to microarray and contamination data. R codes are available from the authors upon request.

## 1. Introduction

While analysing microarray intensity measurements, it is usual to find asymmetric distributions with some negative values and the purpose of this article is to model data with these characteristics.

The traditional approach with microarray data has been to apply transformations that approximately normalize them, with the drawback of losing the original scale. The initial transformation applied was $log_{2}$; it allows working with log-ratios which have a simple and intuitive meaning for biologists (see for example [1,2]). This transformation usually works well for high values but not for zero, and low ones. Further, it cannot be applied to negative values. To avoid these drawbacks, [3,4] suggested the generalized logarithm transformation (*glog*), that allows negative values and this transformation is obtained from a multiplicative–additive linear error model for the data, through a Taylor approximation.

On the other hand, the *glog* transformation usually works well for low values but it is too severe for high ones. The next improvement was introduced by [5], who defined transformations on a real supported data family named generalized power transformations (*gpower*):

(1) $$gpower\left(Y;p\right)=\left\{\begin{array}{cc} \frac{{\left(Y+\sqrt{Y^{2}+1}\right)}^{p}-1}{p} & \mathrm{if}\;p\neq 0\\ \ln\left(Y+\sqrt{Y^{2}+1}\right) & \mathrm{if}\;p=0 \end{array}\right.$$

The *gpower* transformations extend the *glog* transformation continuously, in the same sense as the Box-Cox family [6] extends the natural logarithm.

A related problem presented by microarray data when they are log transformed is the intensity-dependent biases observed in the *MA* (Minus Average) plots (see [7]). These plots display pairwise comparisons of log intensities (Int1 and Int2) between microarrays. More specifically the vertical axis gives the log ratio or difference of the intensities in a log scale $M=log_{2}\left(Int1/Int2\right)=log_{2}\left(Int1\right)-log_{2}\left(Int2\right)$ and the horizontal axis is the average log intensities $A=1/2log_{2}\left(Int1\times Int2\right)=1/2\left(log_{2}\left(Int1\right)+log_{2}\left(Int2\right)\right)$. Even when intensities from a controlled spike-in experiment are expected to show a horizontal cloud centred on the M=0 axis from low to high intensities, non-horizontal structures are apparent. Several approaches have been taken to account for the observed biases (see for example [2,8,9,10]) , explain and correct the observed MA plot intensity-dependent biases through linear transformations.

To avoid transformations, the alternative standpoint taken here is to search for models that fit the data, preserving their scale. One advantage of this strategy is that it facilitates a direct interpretation of the results. In this direction, [11] showed that data that become normal after a *glog* transformation belong to what they called the *glog*-normal distribution family.

In this paper, we extend their results by characterizing those distributions that become normal (or truncated normal) after a *gpower* transformation. We introduce the *gpower*-normal family; this family of distributions should be fitted to gene intensities that have been previously calibrated with an affine transformation, according to the Bengtsson and Hössjer proposal [10].

For positive data, a study has been carried out by [12], who analysed the power normal family, related to Box-Cox transformation. The improvement of the *gpower*-normal family over the *glog*-normal family is that it can account for lighter asymmetries where the *glog* transformation is too strong (see [1,5]); also, it has support on the whole real line, not being restricted to positive values.

In Section 2, we describe the development of a new probability model to be used as a statistical tool for microarrays data analysis. *Gpower*-normal models are defined and their main properties are demonstrated; their relation with pseudo-dispersion models is studied and expressions for the moments and quantiles are obtained. Then, a combined maximum likelihood method is described to obtain estimators of the parameters and it is applied in subsequent sections. For the purpose of illustration, in Section 3, we show several density functions, their quantiles and real data applications. Finally, discussion and conclusions are presented in Section 4 and Section 5 respectively. We have placed some proofs in the Appendices so as not to break the flow of the narrative.

## 2. Methods

The methodological idea within this section is to obtain a model for microarray data in their original scale. With this purpose, we describe a new tool and its implementation.

### 2.1. *Gpower*-normal Distribution

With the goal mentioned above, a new distribution family named *gpower*-normal is presented and its main properties are stated. We are considering data that, when transformed by *gpower*, become normally distributed.

**Definition** **1.** *A random variable Y has a* gpower-*normal distribution if for some* μ∈R*,* σ>0*,* p∈R *its density function is given by*

(2) $$f_{Y}\left(y;\mu ,\sigma^{2},p\right)=\frac{1}{K\sqrt{2\pi \sigma^{2}}}\frac{{\left(y+\sqrt{y^{2}+1}\right)}^{p}}{\sqrt{y^{2}+1}}\exp\left(-\frac{1}{2\sigma^{2}}d_{p}\left(y,\mu \right)\right),\;y\in R$$

*where the normalizing constant* $K=1-\Phi \left(\frac{-1/p-gpower\left(\mu ,p\right)}{\sigma }\right)\;if\;p>0$*,* K=1ifp=0 *and* $K=\Phi \left(\frac{-1/p-gpower\left(\mu ,p\right)}{\sigma }\right)\;if\;p<0$. Φ *is the cumulative distribution function of a standard normal variable and* $d_{p}\left(y,\mu \right)={\left(gpower(y;p)-gpower(\mu ,p)\right)}^{2}$ *is the deviance (see Section 2.2). Density 2 will be denoted as* GPN(μ,σ,p)*, where GPN stands for* gpower-*normal distribution.*

The next theorem gives the main property of *gpower*-normal variables: after a *gpower* transformation they become truncated normals (*TN*). Recall that if *X* is a *TN* variable, its density is given by $f_{X}\left(x,\mu_{X},\sigma^{2}\right)=\frac{1}{K}\frac{1}{\sqrt{2\pi }\sigma }\exp\left(-\frac{1}{2}{\left(\frac{x-\mu_{X}}{\sigma }\right)}^{2}\right)I_{\left(a,b\right)}$, where *I* is the indicator function, $\left(a,b\right)=\left(-1/p,\infty \right)\mathrm{if}\;p>0$, $\left(a,b\right)=\left(-\infty ,\infty \right)\mathrm{if}\;p=0$ and $\left(a,b\right)=\left(-\infty ,-1/p\right)\mathrm{if}\;p<0$; $\mu_{X}\in R$ and we will denote that $X∼TN\left(\mu_{X},\sigma^{2},a,b\right)$ (see Dhrymes [13]).

**Theorem** **1.** *Let* Y∼GPN(μ,σ,p)*, then the transformed variable* $X=gpower\left(Y;p\right)$ *has a truncated normal distribution* $TN\left(\mu_{X},\sigma^{2},-1/p,\infty \right)$ *if* p>0*,* $TN\left(\mu_{X},\sigma^{2},-\infty ,-1/p\right)$ *if* p<0 *and normal distribution* $N\left(\mu_{X},\sigma^{2}\right)$ *if* p=0 *with* $\mu_{X}=gpower\left(\mu ,p\right)$.

**Figure 1:**
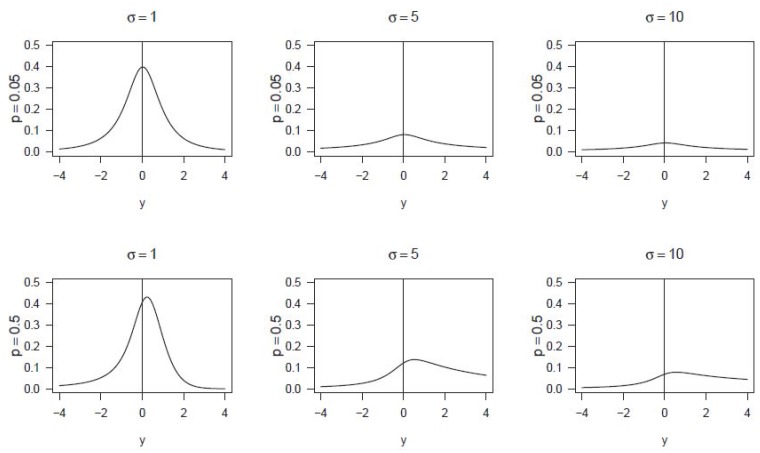
*Gpower*-normal densities for some values of *p* and *σ*, always with *μ* = 0.

The proof of this theorem can be seen in Appendix A. Figure 1 shows the flexibility of this distribution family across different values of the parameters, including symmetric and heavy tailed densities.

### 2.2. Relationship between *Gpower*-normal Models and Pseudo-dispersion Models

*Gpower*-normal models are a special case of a general family of distributions called pseudo-dispersion models defined by [14]. It is proven in Appendix B that the densities defined by (2) belong to this family. Expressions for their deviance and unit variance functions are also obtained. From a theoretical point of view, this is interesting because there are very few examples of pseudo-dispersion models known in the specialized literature.

### 2.3. Quantiles and Moments

A straightforward method to obtain quantiles such as the median and quartiles is considered. These quantiles will be useful in the graphical examination of the model fit to a data set, through quantile–quantile plots.

Let *Y* be a *gpower*-normal random variable and $X=gpower\left(Y;p\right)$ the transformed variable distributed as $TN\left(\mu_{X},\sigma^{2},-1/p,\infty \right)$. Let $x_{\alpha }$ be the *α*-quantile for *X*, to obtain an expression for the quantiles of a truncated normal distribution we proceeded as follows:

$$\begin{array}{cc} P\left(X\leq x_{\alpha }\right) & =P\left(X^{0}\leq x_{\alpha }\right)-F_{X^{0}}\left(-1/p\right)\\ & =\frac{1}{K}\left(\Phi \left(\frac{x_{\alpha }-\mu_{X}}{\sigma }\right)-\Phi \left(\frac{-1/p-\mu_{X}}{\sigma }\right)\right)\\ & =\frac{1}{K}\left(\Phi \left(\frac{x_{\alpha }-\mu_{X}}{\sigma }\right)-\left(1-K\right)\right)=\alpha , \end{array}$$

where $X^{0}$∼$N\left(\mu_{X},\sigma \right)$ is the corresponding normal variable with cumulative distribution $F_{X^{0}}$ and Φ is the cumulative distribution of the standard normal. Now, clearing up

$$x_{\alpha }=\sigma \Phi^{-1}\left(K(\alpha -1)+1\right)+\mu_{X}.$$

Also

$$\alpha =P\left(X\leq x_{\alpha }\right)=P\left(gpower\left(Y\right)\leq x_{\alpha }\right)=P\left(Y\leq gpower^{-1}\left(x_{\alpha }\right)\right)$$

then the *α*-quantile for *Y* is

(3) $$y_{\alpha }=gpower^{-1}\left(x_{\alpha }\right)$$

and its value can be obtained from the standard normal distribution. This procedure will be applied in Section 3.2.

Expressions for the moments of a *gpower*-normal family can be expressed in terms of the truncated normal density function (see Appendix C). However, these expressions are not easy to handle, and their convergence for different values of the parameters remains to be analysed. In order to avoid the difficulties in moment calculations (e.g., mean and variance) for models given in Definition 1, we propose the alternative use of quantiles.

### 2.4. Parameter Estimation

The *gpower*-normal models have three parameters to be estimated. They are related to the corresponding TN model parameters as it has been detailed in Section 2.1. We propose a combined profile likelihood and maximum likelihood approach to estimate the parameters. The five steps of the proposed estimation approach are described below:

1. Given a data set represented by vector $y=(y_{1}$, $y_{2}$, …, $y_{n})$, to obtain a profile likelihood for the power *p*, we consider a grid of values $p_{0}$, $p_{1}$, …, $p_{k}$.
2. For each $p_{j}$, 1≤j≤k the transformed data $x_{p_{j}}$ are calculated as $x_{p_{j}}=gpower\left(y,p_{j}\right).$
3. Then, for each $p_{j}$, the corresponding $\mu_{p_{j}}$ and $\sigma_{p_{j}}$ are estimated, maximizing the likelihood function of the truncated normal variable.
4. Then, $p_{j}$, $\mu_{p_{j}}$ and $\sigma_{p_{j}}$ are used to obtain the log-likelihood function of y whose density was given by (2):
  $$\ln f_{y}\left(y;\mu ,\sigma^{2},p\right)=n\ln\frac{1}{K\sqrt{2\pi \sigma_{p_{j}}^{2}}}+\sum_{i=1}^{n}\left(\ln p\left(y_{i}+\sqrt{y_{i}^{2}+1}\right)-\ln\sqrt{y_{i}^{2}+1}\right)-\frac{1}{2\sigma^{2}}\sum_{i=1}^{n}d_{p}\left(y_{i},\mu_{p_{j}}\right).$$
5. Finally, *p* is chosen as the one that maximizes the log-likelihood in the grid: $\hat{p}=\max_{1\leq j\leq k}\ln f_{y}\left(y;\mu_{p_{j}},\sigma_{p_{j}}^{2},p_{j}\right)$

The method described above is applied in Section 3.2 and an implementation has been written in R language [15]. The codes are available from the authors upon request.

## 3. Results

To highlight the potential of the distribution family to model data with different skewness and kurtosis, we present several density functions and their quantiles. Also, the model fit is illustrated with real data sets.

### 3.1. Some Examples of *Gpower*-normal Densities

By way of example, Figure 1 shows the flexibility of this distribution family across different values of the parameters, including symmetric and heavy tailed densities. Graphic representations of these densities are exhibited for two different values of *p* (0.05 and 0.5) and three different values of *σ* (1, 5 and 10); always with μ=0.

It can be seen that kurtosis decreases as *σ* grows up and asymmetry grows with *p*.

Table 1 contains some quantile values corresponding to the distributions displayed in Figure 1 confirming the observations made above for those displays.

### 3.2. Real Data Applications

As it was mentioned in Section 1, the proposed density family was originally motivated by the modelling of microarray intensities, but its application is more general. Here, we present some examples: the first one corresponds to intensities of microarray data, the second one to concentrations of ammonia and the third one to magnetic contamination indices. For these examples, the parameter estimators are obtained by the method described in Section 2.4. Then, the data fit to the corresponding estimated model is shown graphically in three ways: (1) by the overlap of the data histograms with the density curves, (2) by the overlap of the empirical distribution curve with the adjusted model cumulative distribution function, and (3) by quantile–quantile (Q–Q) plots, that display the ordered data in the horizontal axis and the corresponding quantiles of the estimated model distribution.

It is also evaluated applying Kolmogorov–Smirnov tests to compare the estimated *gpower*-normal and the *glog*-normal distributions. The resulting *p*-values should be taken as descriptive comparisons of the two distributions and as a complement of the Q–Q plots.

**Example** **1.** The first set of data corresponds to 30 intensities of one gene and they can be seen in Appendix D. These data were selected from the Yale University MAQC project and downloaded from [16].

**Figure 2:**
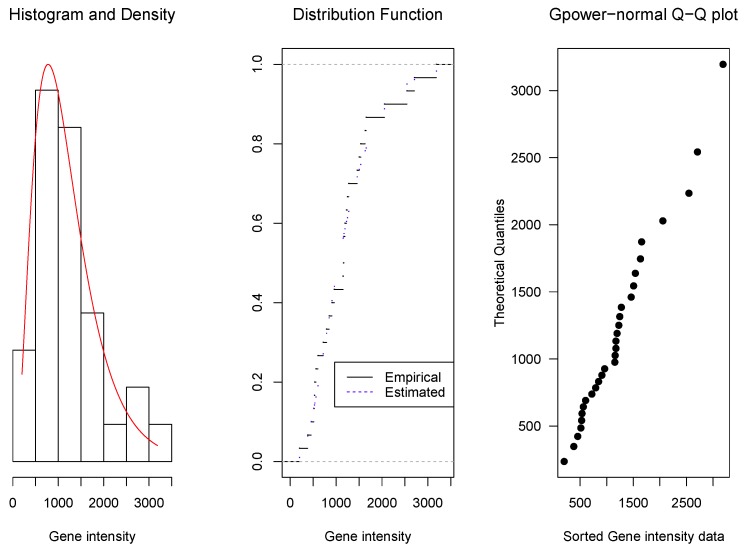
Overlap of the histogram with the adjusted model density curve (**left**), of the empirical distribution curve with the distribution function (**center**) and quantile–quantile (Q–Q) plot (**right**) for gene expression data.

*The values for* p, *μ, and σ were estimated by the profile likelihood method (Section 2.4), resulting* $\hat{p}$ = *0.23,* $\hat{\mu }$ = *1052 and* $\hat{\sigma }$ = *3.46. From these estimations, the 0.25, 0.50 and 0.75 quantiles were obtained according to expression (3) resulting in 690, 1052 and 1545, respectively. As can be seen in Figure 2, this set of data is well fitted by the* gpower-*normal model with the parameters given above. It is confirmed by the Kolmogorov–Smirnov test, with a p-value = 0.7057 for the* gpower-*normal model and 0.4934 for the* glog-*normal model.*

**Example** **2.** *Two sets of data representing concentrations of ammonia in the UK for the years 2005 and 2011 were downloaded from [17] and they are given in Appendix D. Applying again the methodology detailed in Section 2.4, we obtained, for the year 2005, an optimum value of 0.20 for the parameter* p. *The corresponding, μ, σ and the 0.25, 0.50 and 0.75 quantiles were 2.11, 0.70 , 1.39, 2.11 and 3.04 respectively. For the year 2011, the optimum* p *was 0.16 and the corresponding* $\hat{\mu }$, $\hat{\sigma }$ *and the 0.25, 0.50 and 0.75 quantiles were 2.93, 0.68, 2.00, 2.93 and 4.15 respectively. Observe that the 2011 interquartile interval shows a level and amplitude increase with respect to 2005.*

**Figure 3:**
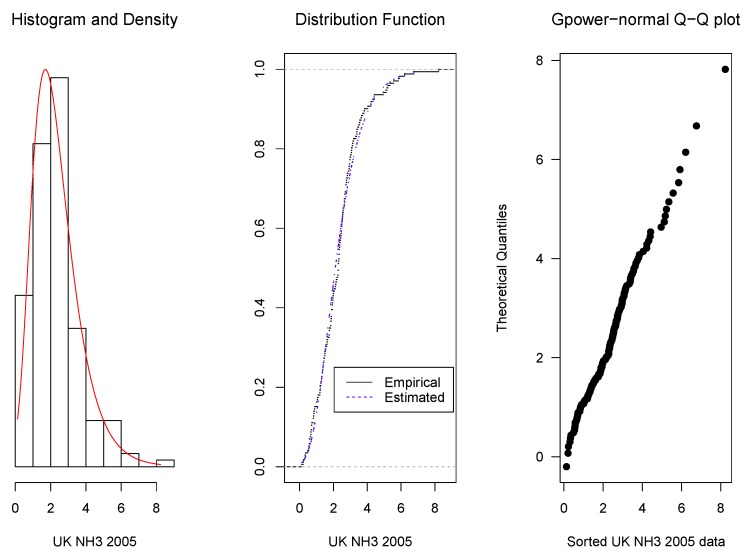
Overlap of the histogram with the adjusted model density curve (**left**), of the empirical distribution curve with the distribution function (**center**) and Q–Q plot (**right**) for concentrations of ammonia in 2005.

**Figure 4:**
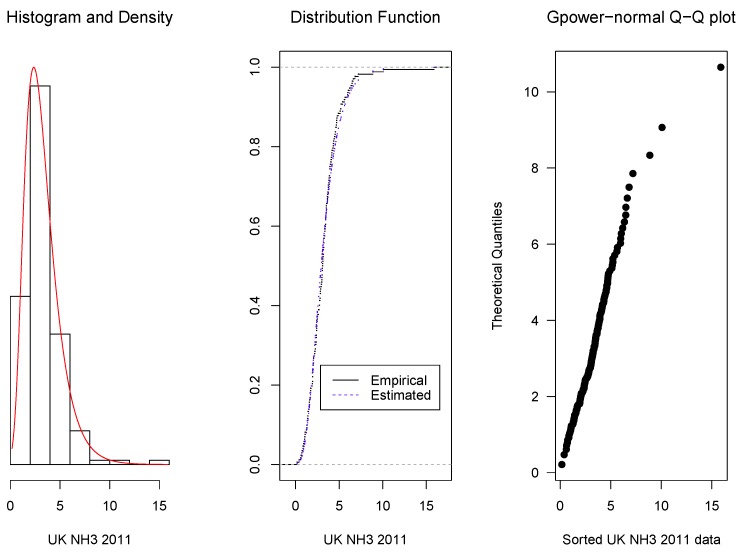
Overlap of the histogram with the adjusted model density curve (**left**), of the empirical distribution curve with the distribution function (**center**) and Q–Q plot (**right**) for concentrations of ammonia in 2011.

*As in the previous example, it can be seen in Figure 3 and Figure 4 that the fitted* gpower-*normal models with the parameters given above fit them quite well.*

*In the Q–Q plot for the year 2011 data, the highest three observations are higher than the expected model quantiles, suggesting an increase in ammonia concentration. The Kolmogorov–Smirnov test confirms the goodness-of-fit with a significance of 0.3411 for 2005 and 0.4993 for 2011. For the corresponding* glog-*normal model, p-values of 0.1168 and 0.2327 were obtained.*

**Example** **3.** *When measuring magnetic contamination, the index can take an asymmetric positive distribution, with some negative values observed because of air presence. In this example, data from Mar del Plata, Argentina (see [18]), are fitted with a* gpower-*normal density. Data can be seen in Appendix D. Applying again the methodology detailed in Section 2.4, we obtained an optimum value of 0.19 for the parameter* p. *The corresponding* $\hat{\mu }$, $\hat{\sigma }$ *and the 0.25, 0.50 and 0.75 quantiles were 8.05, 1.64 , 3.98, 8.05 and 14.91 respectively.*

**Figure 5:**
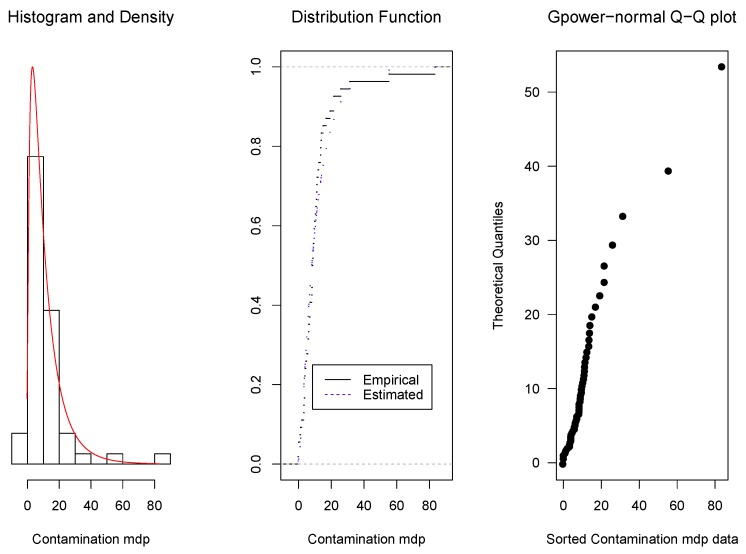
Overlap of the histogram with the adjusted model density curve (**left**), of the empirical distribution curve with the distribution function (**centre**) and Q–Q plot (**right**) for magnetic contamination in Mar del Plata.

*As in the previous examples, it can be seen in Figure 5 that the fitted* gpower-*normal model with the parameters given above fits them quite well. The fit was evaluated applying a Kolmogorov–Smirnov test (p-value = 0.57 for the* gpower-*normal model and 0.29 for the* glog-*normal model).*

## 4. Discussion

A new family of distributions named *gpower*-normal, indexed by p∈R, has been defined. Variables whose distribution belongs to this family become normal or truncated normal when a *gpower* transformation is applied. From a practical point of view, the truncation is very often negligible, such is the case of data in Example 1. The estimated parameters were $\hat{p}=0.23$ , $\hat{\mu }=1052$ and $\hat{\sigma }=3.516$ and thus the truncation constant given in Definition 1 is almost 1, meaning that no truncation is necessary in this case. Similar results were obtained for Examples 2 and 3.

The *gpower*-normal family can model data that include non-positive values, as required for microarray analysis.

To estimate the model parameters, a combined maximum likelihood method is proposed and it is successfully applied to real data. It enables direct calculations of quantiles for which simple expressions are given. Thus, position and scale measures (i.e., the median and the interquartile range) can be easily obtained, overcoming the difficulties found in moment calculations.

This allows the use of the estimated distribution medians as summary measures for gene intensities. Therefore, to compare the gene intensities in two biological conditions, it seems adequate to compare the corresponding medians of the estimated distributions.

This paper extends the results previously presented by Leiva and coauthors [11] and therefore the new distribution family offers a larger set of models. Considering the Leiva et al. proposal as a standard alternative, the new family can fit data for which their proposal might be not flexible enough. As expected, in all examples, the Kolmogorov–Smirnov tests indicated a closer fit of the data to the *gpower*-normal distribution in comparison with the *glog*-normal fit.

In addition, it has been proven that the *gpower*-normal family is a special case of pseudo-dispersion models, inheriting all the good properties of these models, such as asymptotic normality for small variances. It should be pointed out that very few examples of pseudo-dispersion models have been reported in the literature. The obtained *p*-values should be considered as descriptive values only and as a complement to the Q–Q plot, which provides a visual impression of the fit.

## 5. Conclusions

We have presented a family of distributions to model asymmetric data with positive and negative values. It allows investigators to model microarray data, preserving their original scale. Although the construction of this family of distributions was motivated by microarray data, it is important to notice that the model is also useful in other environments, such as chemical and magnetical contamination data. A method has been given to estimate quantiles and in our examples, specific quantile–quantile plots were effective tools for a visual evaluation of the data fit to the estimated models.

## Figures and Tables

**Table 1:** Some *α* quantile values for different choices of the parameters in Expression 2: p=0.05,0.50, σ=1,5,10 always with μ=0.

		*p* = 0.05			*p* = 0.50	
*α*	*σ* = 1	*σ* = 5	*σ* = 10	*σ* = 1	*σ* = 5	*σ* = 10
0.01	−11.78	−3.65 × 7	−1.63 × 22	−166.37	−508.68	−182.81
0.05	−5.39	−3.98 × 10^4^	−2.73 × 10^11^	−13.84	−20.83	−7.25
0.10	−3.49	−2.26 × 10^3^	−4.64 × 10^7^	−5.67	−5.17	−1.33
0.50	0.00	3.97 × 10^−4^	0.57	0.06	4.25	14.16
0.90	3.17	259.00	2.17 × 10^4^	2.34	22.75	78.52
0.95	4.65	981.00	1.74 × 10^5^	3.04	31.37	109.85
0.99	8.92	9590.00	5.24 × 10^6^	4.48	51.85	185.57

## Appendix A. Proof of Theorem 1

In this Theorem, it is shown that a *gpower*-normal variable becomes normal or truncated normal when its corresponding *gpower* transformation is applied.

**Proof.** Let $x=gpower\left(y;p\right)$, then when p≠0

$$y=gpower^{-1}\left(x\right)=\frac{{\left(px+1\right)}^{1/p}-{\left(px+1\right)}^{-1/p}}{2}$$

and it is easy to see that

$$\frac{\partial y}{\partial x}=\frac{\partial \;gpower^{-1}\left(x\right)}{\partial x}\;=\frac{{\left(px+1\right)}^{1/p}+{\left(px+1\right)}^{-1/p}}{2(px+1)}$$

for p>0, x>−1/p and the derivative is positive, which means that $gpower\left(y;p\right)$ is a monotonically increasing function. Now, $X=gpower\left(Y\right)$ is considered as a function of a random variable *Y*∼GPN(μ,σ,p). The density for *X* is obtained as follows:

$$\begin{array}{cc} f_{X}\left(x\right) & =\frac{\partial }{\partial x}P\left(gpower\left(Y;p\right)\leq x\right)\\ & =f_{Y}\left(\frac{{\left(px+1\right)}^{1/p}-{\left(px+1\right)}^{-1/p}}{2}\right)\frac{{\left(px+1\right)}^{1/p}+{\left(px+1\right)}^{-1/p}}{2(px+1)}\\ & =\frac{1}{K\sqrt{2\pi \sigma^{2}}}\exp\left(-\frac{1}{2\sigma^{2}}\;\left(x-\mu_{X}{)}^{2}\right)\right),\;-1/p<x<\infty \end{array}$$

that is precisely the density of a $TN\left(\mu_{X},\sigma^{2},-1/p,\infty \right)$ random variable. For p<0, it can be proved analogously that

$$f_{X}\left(x\right)=\frac{1}{K\sqrt{2\pi \sigma^{2}}}I_{\left(-\infty ,-1/p\right)}\left(x\right)\exp\left(-\;\frac{1}{2\sigma^{2}}{\left(x-\mu_{X}\right)}^{2}\right),$$

For p=0, $gpower\left(x,0\right)=\ln\left(y+\sqrt{y^{2}+1}\right)=arc\sinh\left(y\right)$. The inverse transformation is $gpower^{-1}\left(x,0\right)=\sinh\left(x\right)=\frac{e^{x}-e^{-x}}{2}$ and

$$\frac{\partial gpower^{-1}\left(x,0\right)}{\partial x}=\frac{\partial }{\partial x}\left(\frac{e^{x}-e^{-x}}{2}\right)=\frac{e^{x}+e^{-x}}{2}.$$

Again, the derivative is positive ∀y, meaning that the function is monotonically increasing and the density of *X* becomes

$$\begin{array}{cc} f_{X}\left(x\right) & =\frac{d}{dy}P\left(Y\leq \frac{e^{x}-e^{-x}}{2}\right)\frac{dy}{dx}=\frac{1}{\sqrt{2\pi \sigma^{2}}}\exp\left(-\frac{1}{2\sigma^{2}}\;\left(x-\mu_{X}{)}^{2}\right)\right) \end{array}$$

that is precisely the density of a $N\left(\mu_{X},\sigma^{2}\right)$ random variable.  ☐

## Appendix B. Relationship between *Gpower*-normal Models and Pseudo-dispersion Models

We first define proper dispersion models and then extend them to pseudo-dispersion models.

**Definition** **2.** *Given an open interval* Ω⊆R*, a proper dispersion model is a family of random variables Y with density functions of the form*

$$f_{Y}\left(y;\mu ,\sigma^{2}\right)=c\left(\sigma^{2}\right)V^{-1/2}\left(y\right)\exp\left(-\frac{1}{2\sigma^{2}}d\left(y,\mu \right)\right),y\in \Omega$$

*where* μ∈Ω*,* σ>0*,* $d\left(y,y\right)=0$ ∀y∈Ω *and* $d\left(y,\mu \right)>0$ ∀y≠μ *(which means that d is a deviance). The constant* $c\left(\sigma^{2}\right)$ *ensures that this is a density function and* $V\left(\right)$ *is the variance function, related with d by* $V\left(y\right)=-\frac{2}{{\left.\frac{\partial^{2}d\left(y,\mu \right)}{\partial y\partial \mu }\right|}_{y=\mu }}$.

Proper dispersion models are characterized by the existence of vector functions s and ***α*** such that their deviance can be factorized as $d\left(y,\mu \right)=s{\left(y\right)}^{T}\alpha \left(\mu \right)$.

The above definition can be extended, by allowing $c\left(\right)$ todepend also on *μ* and the new density is

(4) $$f_{Y}\left(y,\mu ,\sigma^{2}\right)=c\left(\mu ,\sigma^{2}\right)V^{-1/2}\left(y\right)\exp\left(-\frac{1}{2\sigma^{2}}d\left(y,\mu \right)\right),\;y\in \Omega$$

being

$$\frac{1}{c\left(\mu ,\sigma^{2}\right)}=\int_{\Omega }V^{-1/2}\left(y\right)\exp\left(-\frac{1}{2\sigma^{2}}d\left(y,\mu \right)\right)dy.$$

On the other hand, if it satisfies that $\sigma c\left(\mu ,\sigma^{2}\right)⟶{\left(2\pi \right)}^{-1/2},\;\mathrm{when}\;\sigma \to 0$ for all *y*, it is called a pseudo-dispersion model. A remarkable property of these models is that they are asymptotically normal [14].

It will be proven now that *gpower*-normal models are pseudo-dispersion models.

**Theorem** **2.** Models given in Definition 1 are pseudo-dispersion models.

**Proof.** First note that ${\left(gpower\left(y;p\right)-gpower\left(\mu ,p\right)\right)}^{2}$ is a deviance. Besides, defining $V\left(y\right)=\left(y^{2}+1\right)/{\left(y+\sqrt{y^{2}+1}\right)}^{2p}$, equality given in 2 is verified and the densities given in (2) can be written as (4) where the normalizing constant is

$$c\left(\mu ,\sigma^{2}\right)=\frac{1}{\left(1-\Phi \left(\frac{-1/p-gpower\left(\mu \right)}{\sigma }\right)\right)\sqrt{2\pi }\sigma }$$

To verify that it satisfies the limit condition, it has to be seen that $\sigma c\left(\mu ,\sigma^{2}\right)\to \frac{1}{\sqrt{2\pi }}$ when σ→0. This is true because $\frac{-1/p-gpower\left(\mu \right)}{\sigma }\underset{\sigma \to 0}{⟶}-\infty$ and $\Phi \left(-\infty \right)=0$.  ☐

Besides, note that it is enough to define $s{\left(y\right)}^{T}=\left(gpower^{2}\left(y;p\right),-\sqrt{2}gpower\left(y;p\right),1\right)$ and $\alpha {\left(\mu \right)}^{T}=\left(1,\sqrt{2}gpower\left(\mu ,p\right),gpower^{2}\left(\mu ,p\right)\right)$ to be able to express $d_{p}\left(y,\mu \right)$ as $s{\left(y\right)}^{T}\alpha \left(\mu \right)$. In particular, if p=0, the variance function is $V\left(\mu \right)=\mu^{2}+1$ and it coincides with the variance function of a generalized hyperbolic secant, that corresponds to a Morris model [19].

## Appendix C. Moments of *Gpower*-normal Family

Expressions for the moments of a *gpower*-normal family are obtained here, and it is shown that they can be expressed in terms of the truncated normal density function.

**Theorem** **3.** *Let* Y∼GPN(μ,σ,p)*, then its moments can be expressed as* $E\left(Y^{n}\right)=\frac{1}{2K\sqrt{2\pi \sigma^{2}}}\int_{-1/p}^{\infty }{\left({\left(px+1\right)}^{1/p}-{\left(px+1\right)}^{-1/p}\right)}^{n}exp\left(-\frac{1}{2}{\left(\frac{x-\mu_{X}}{\sigma }\right)}^{2}\right)dx$.

**Proof.** *Y* can be represented as the inverse *gpower* transformation of a truncated normal variable *X*:

$$Y=gpower^{-1}\left(X,p\right)=\left\{\begin{array}{cc} \frac{{\left(pX+1\right)}^{1/p}-{\left(pX+1\right)}^{-1/p}}{2} & \mathrm{if}\;p\neq 0\\ \frac{e^{X}-e^{-X}}{2} & \mathrm{if}\;p=0 \end{array}\right.$$

now, the expectation of $Y^{n}$ can be expressed for p≠0 as

$$\begin{array}{cc} E\left(Y^{n}\right) & =E{\left(\frac{{\left(pX+1\right)}^{1/p}-{\left(pX+1\right)}^{-1/p}}{2}\right)}^{n}\\ & =\frac{1}{2^{n}K\sqrt{2\pi \sigma^{2}}}\int_{-1/p}^{\infty }{\left({\left(px+1\right)}^{1/p}-{\left(px+1\right)}^{-1/p}\right)}^{n}exp\left(-\frac{1}{2}{\left(\frac{x-\mu_{X}}{\sigma }\right)}^{2}\right)dx. \end{array}$$

☐

## Appendix D. Data used in Examples

### Example 1

1276, 910, 1159, 561, 1174, 1174, 2707, 2056, 1539, 962, 1635, 604, 848, 1506, 1195, 793, 1657, 3187, 1246, 2546, 537, 456, 201, 1153, 515, 1228, 529, 1461, 382, 721.

### Example 2

2005: 3.16 6.21 5.17 5.57 4.96 2.58 1.01 1.43 1.27 1.87 0.71 0.63 0.67 3.06 0.49 1.85 1.94 0.33 1.34 2.73 2.45 2.71 3.57 2.30 1.39 2.52 2.17 2.54 2.46 1.89 2.06 2.50 2.26 2.97 3.50 1.89 1.97 3.07 3.39 3.08 4.33 8.23 5.35 5.85 4.04 3.81 3.44 5.23 5.92 4.22 2.67 1.55 1.50 1.95 1.43 3.40 2.38 2.96 1.36 0.13 0.21 0.69 1.96 0.23 0.55 2.13 3.55 4.40 2.73 5.12 3.64 3.85 2.12 2.49 2.33 2.53 2.64 4.42 3.67 3.13 2.64 3.02 2.92 2.73 2.97 2.36 3.74 2.16 2.76 3.12 3.33 1.84 2.29 2.63 2.54 2.41 2.98 2.47 2.34 2.29 2.57 2.67 2.32 2.28 1.40 1.76 2.79 2.34 2.55 2.82 2.29 2.22 1.63 0.54 0.31 1.01 1.62 0.71 1.94 1.47 0.36 0.57 1.61 0.81 2.43 2.65 1.20 1.38 1.26 1.85 1.54 1.31 0.89 0.83 0.85 1.30 1.75 1.34 1.76 2.83 2.00 1.22 1.51 1.88 2.37 2.88 3.20 3.38 4.23 2.01 1.06 1.05 1.77 1.22 0.90 2.77 1.21 1.89 0.84 0.72 1.11 0.81 0.66 2.28 2.33 2.49 2.93 1.67 1.98 0.58 6.76 0.58.
2011: 3.25 6.82 7.20 6.49 5.40 4.09 2.84 2.28 2.08 3.25 1.57 1.06 0.75 3.44 0.86 2.37 2.45 0.63 1.67 3.43 3.45 4.29 4.47 2.77 1.95 3.12 2.77 3.47 4.19 2.73 3.76 3.92 4.17 6.19 4.37 2.46 2.82 3.65 4.71 3.50 6.50 15.89 8.87 10.08 5.97 6.36 4.61 5.99 6.64 4.69 3.06 1.66 2.02 1.73 1.35 3.20 2.02 2.73 1.43 0.17 0.40 1.43 2.36 0.87 1.24 2.88 4.15 4.78 4.74 6.05 3.92 3.98 3.67 2.83 3.14 3.54 3.01 5.22 4.56 3.94 3.27 3.81 3.82 3.92 4.33 3.45 5.12 3.64 4.42 5.16 5.67 2.90 5.22 4.70 4.32 3.70 3.88 3.35 3.02 3.06 3.18 3.55 3.10 3.22 1.93 2.19 3.52 3.15 3.33 3.85 2.83 2.97 2.49 1.55 0.61 1.59 2.26 1.97 4.11 2.55 1.67 2.38 2.05 2.22 3.05 3.75 3.09 1.96 2.09 2.64 2.40 2.01 1.50 1.35 1.41 1.96 2.46 1.97 2.50 3.52 2.64 1.80 2.08 2.37 3.77 4.05 4.61 4.91 5.61 3.20 1.38 3.36 3.35 1.60 1.09 3.48 1.71 2.40 0.99 1.04 1.30 1.10 0.93 3.00 3.08 3.27 3.66 2.28 2.42 0.72 4.54 1.29.

### Example 3

8.8 0.9 10.2 25.9 21.5 8.3 12.4 3.9 11.4 3.2 11.1 5.8 6.7 13.8 83.4 8.3 6.2 8.9 19.2 13.5 15.0 31.3 8.2 5.0 3.9 11.2 8.3 12.0 9.5 5.8 6.3 14.0 21.5 10.6 7.1 9.5 9.0 3.9 3.5 7.1 16.9 4.0 4.5 10.9 55.3 3.3 1.3 13.4 -0.3 2.0 0.0 9.8 0.0 8.1
